# The first Loranthaceae fossils from Africa

**DOI:** 10.1080/00173134.2018.1430167

**Published:** 2018-02-15

**Authors:** Friðgeir Grímsson, Alexandros Xafis, Frank H. Neumann, Louis Scott, Marion K. Bamford, Reinhard Zetter

**Affiliations:** 1 Department of Palaeontology, University of Vienna, Vienna, Austria; 2 School of Agricultural, Earth and Environmental Sciences, University of KwaZulu-Natal, Pietermaritzburg, South Africa; 3 Department of Plant Sciences, University of the Free State, Bloemfontein, South Africa; 4 Evolutionary Studies Institute, University of the Witwatersrand, Johannesburg, South Africa

**Keywords:** Santalales, diagnostic pollen, host plants, Miocene, palaeoecology, palaeophytogeography, parasitic plants, pollen morphology

## Abstract

An ongoing re-investigation of the early Miocene Saldanha Bay (South Africa) palynoflora, using combined light and scanning electron microscopy (single grain method), is revealing several pollen types new to the African fossil record. One of the elements identified is Loranthaceae pollen. These grains represent the first and only fossil record of Loranthaceae in Africa. The fossil pollen grains resemble those produced by the core Lorantheae and are comparable to recent Asian as well as some African taxa/lineages. Molecular and fossil signals indicate that Loranthaceae dispersed into Africa via Asia sometime during the Eocene. The present host range of African Loranthaceae and the composition of the palynoflora suggest that the fossil had a range of potential host taxa to parasitise during the early Miocene in the Saldanha Bay region.

The Loranthaceae is a large family with *c*. 76 genera and at least 1000 species divided into five tribes (Nickrent 1997–onwards; Nickrent et al. 2010). The family is widely distributed and occurs in tropical to temperate regions of Australasia, Asia, the Middle East, Africa, Europe, and Central and South America (e.g. Barlow 1983; Polhill & Wiens 1998), showing a clear geographic split between a New World group (Psittacanthinae Engl.) and Old World-Australasian lineages (Elythrantheae Engl. and Lorantheae Rchb.; e.g. Nickrent et al. 2010; Grímsson et al. 2017, 2018). The *c*. 238 species and 21 genera occurring in Africa (Table I) are considered to be the most derived in the family. Most of the African genera/species are endemic, with only *Helixanthera* and *Taxillus* extending into Asia. *Helixanthera*, occurring from Africa to Indonesia, is regarded as the most primitive Lorantheae genus thriving in continental Africa (Polhill & Wiens 1998). Even though Loranthaceae are currently found all over Africa (except the Sahara desert), it has been suggested that they dispersed to the continent during the Cretaceous (Gondwanan derivation) or Eocene times (Asian derivation) (Barlow 1983, 1990; Polhill & Wiens 1998), however, no fossil Loranthaceae have ever been reported from this part of the world. The fossil record of Loranthaceae, recently summarised by Grímsson et al. (2017, figures 10, 11, and file S4), shows that the family already had a global distribution during the Eocene, occurring on all continents except Africa and Antarctica. The Loranthaceae have a fragmentary fossil record composed solely of fossil pollen (Grímsson et al. 2017), most likely due to the ecology and life cycle of Loranthaceae (small woody plants, with relatively few leaves, their fruits are ingested by birds, and seeds germinate immediately after regurgitation; see Polhill & Wiens 1998) . Therefore, the only way to trace the origin and evolution of this family, in time and space, is to study fossil Loranthaceae pollen in relation to phylogeny. Grímsson et al. (2018) evaluated the correlation of pollen morphology and molecular phylogenetic relationships within Loranthaceae and discovered that most pollen types in this family are linked to a single genus or discrete evolutionary lineages. Since pollen types produced by most extant members of the Loranthaceae are distinct (Feuer & Kuijt 1978, 1979, 1980, 1985; Kuijt 1988; Liu & Qiu 1993; Han et al. 2004; Roldán & Kuijt 2005; Caires 2012; Caires et al. 2012, 2014, 2017; Grímsson et al. 2017, 2018) and cannot be confused with pollen from other related families, fossil Loranthaceae pollen give the potential to trace modern lineages back in time.

**Table I.** African Loranthaceae genera and their hosts.

Genus	Number of species	African species	Occurrence in Africa	Recorded host families in Africa	See Table
*Helixanthera*	*c*. 45	12	Tropical, scattered around edge of continent	**Anacardiaceae**, Bignoniaceae, Boraginaceae, Burseraceae, **Combretaceae**, **Euphorbiaceae**, **Fabaceae**, Lauraceae*, **Loranthaceae**, Malvaceae*, Moraceae, Ochnaceae, Phyllanthaceae, Rhamnaceae, Rubiaceae*, Rutaceae, **Sapotaceae**	S1
*Plicosepalus*	12	12	Eastern side of Africa to Angola and South Africa	**Anacardiaceae**, Apocynaceae, Burseraceae, **Combretaceae**, **Fabaceae**, Rubiaceae	S2
*Emelianthe*	1	1	Drier parts of E. and NE. Africa	**Anacardiaceae**, Burseraceae, **Euphorbiaceae**, Malvaceae	S3
*Pedistylis*	1	1	Southern Africa	**Anacardiaceae**, **Combretaceae**, Ebenaceae, **Fabaceae**, Meliaceae, Moraceae	S4
*Actinanthella*	2	2	SE. and S. Africa	Capparaceae, Erythroxylaceae, **Oleaceae**	S5
*Oncocalyx*	13	13	Drier forests and bushland of eastern and southern Africa	**Anacardiaceae**, Apocynaceae, Boraginaceae, Burseraceae, Cannabaceae, Capparaceae, Celastraceae, **Combretaceae**, Ebenaceae, **Euphorbiaceae**, **Fabaceae**, Malvaceae, Pittosporaceae, Rhamnaceae, Salicaceae, Salvadoraceae, Tamariaceae, Zygophyllaceae	S6
*Spragueanella*	2	2	E. and SC. Africa along coast and extending into mountains in dryer forest	**Podocarpaceae**, Putranjivaceae	S7
*Oliverella*	3	3	Eastern and south-central Africa in coastal and deciduous bushland and mixed woodland	**Combretaceae**, **Euphorbiaceae**, **Fabaceae**, Malvaceae	S8
*Berhautia*	1	1	Senegal and Gambia	**Combretaceae**	S9
*Englerina*	25	25	Tropical Africa	Achariaceae, **Asteraceae**, Bignoniaceae, Boraginaceae, Buddlejaceae, Burseraceae, Clusiaceae, **Combretaceae**, Ebenaceae, **Fabaceae**, Loganiaceae, Malvaceae, **Oleaceae**, Primulaceae, **Proteaceae**, Rhamnaceae, Rubiaceae, Rutaceae*, **Sapindaceae**	S10
*Agelanthus*	59	59	Africa south of the Sahara	**Anacardiaceae**, Apocynaceae, **Asteraceae**, Boraginaceae, Burseraceae, Cannabaceae, Cappaeaceae, Celastraceae, Chrysobalanaceae, **Combretaceae**, Convolvulaceae, Erythroxylaceae, **Euphorbiaceae**, **Fabaceae**, Iteaceae, Juglandaceae*, Lamiaceae, **Loranthaceae**, Lythraceae*, Malvaceae, Meliaceae, Moraceae, Olacaceae, **Oleaceae**, Phyllanthaceae, Plumbaginaceae, Rhamnaceaee, Rosaceae*, Rutaceae*, **Proteaceae**, Salicaceae, Salvadoraceae, **Santalaceae**, **Sapindaceae**, Solanaceae*, Ulmaceae, Urticaceae, **Vitaceae**	S11
*Tapinanthus*	30	30	Tropical and southern Africa	**Anacardiaceae**, Apocynaceae, Asphodelaceae, **Asteraceae**, Burseraceae, Celastraceae, **Combretaceae**, Crassulaceae, Ebenaceae, **Euphorbiaceae**, **Fabaceae**, Juglandaceae*, Kirkiaceae, Lamiaceae, **Loranthaceae**, Malvaceae, Meliaceae, Melianthaceae, Moraceae, **Myrtaceae**, Ochnaceae, Phyllanthaceae, **Proteaceae**, Rosaceae, Rhamnaceae, Rutaceae, Salicaceae, Salvadoraceae, **Santalaceae**, **Sapotaceae**, Solanaceae, Tamaricaceae	S12
*Moquiniella*	1	1	Southern Namibia and the Cape Province of South Africa	**Anacardiaceae**, Apocynaceae, Ebenaceae, **Fabaceae**, Hypericaceae, Malvaceae, Moraceae, Rosaceae*, Salicaceae	S13
*Globimetula*	13	13	Tropical Africa	Anacardiaceae*, Burseraceae, Chrysobalanaceae, **Combretaceae**, **Fabaceae**, Malvaceae*, Meliaceae, Moraceae, Myrtaceae*, Phyllanthaceae, **Proteaceae**, Rutaceae*	S14
*Taxillus*	35	1	Coast of Kenya	**Fabaceae**	S15
*Vanwykia*	2	2	Eastern and south-eastern Africa	**Fabaceae**, Moraceae	S16
*Septulina*	2	2	Western Cape Province of South Africa and southern Namibia	Aizoaceae, **Anacardiaceae**, **Fabaceae**, Solanaceae, Tamaricaceae	S17
*Oedina*	4	4	Montane forests from Tanzania to northern Malawi		S18
*Oncella*	4	4	Montane and coastal areas of eastern Africa	Phyllanthaceae, Malvaceae, Meliaceae	S19
*Erianthemum*	16	16	Eastern and southern Africa	**Anacardiaceae**, Archariaceae, **Asteraceae**, Bignoniaceae*, Burseraceae, Celastraceae, **Combretaceae**, Ebenaceae, **Euphorbiaceae**, **Fabaceae**, Lamiaceae, Loganiaceae, Malvaceae, Meliaceae, Myrtaceae*, Phyllanthaceae, **Proteaceae**, Rhamnaceae, Rosaceae*, Rutaceae*, **Sapotaceae**	S20
*Phragmanthera*	34	34	Tropical forests of Africa, few extend into dry habitats in south-central and southern Africa	Anacardiaceae*, Annonaceae, Boraginaceae, Burseraceae, Casuarinaceae*, **Combretaceae**, **Euphorbiaceae**, **Fabaceae**, Irvingiaceae, Lauraceae*, Malvaceae*, Melianthaceae, Moraceae, Myrtaceae*, Rhamnaceae, Rubiaceae, Rutaceae*, Tamaricaceae, Phyllanthaceae, **Proteaceae**, **Sapotaceae**	S21

Notes: Families with introduced host taxa are marked with asterisk*. Host families known from the fossil palyno-assemblage appear in **bold.** Loranthaceae systematics and distribution summarised from Polhill and Wiens (1998), data on host taxa compiled from Wiens and Tölken (1979), Visser (1981), Dean et al. (1994), Polhill and Wiens (1998, 1999), Dzerefos et al. (2003), Roxburgh and Nicolson (2005), Veste (2007), Didier et al. (2008), Ogunmefun et al. (2013), Dlama et al. (2016) and Okubamichael et al. (2013, 2016). See also Tables S1–S21 in Supplemental data.

Here we describe a new fossil Loranthaceae pollen type from the earliest Miocene of Saldanha Bay, South Africa. These fossils are the first representatives of this family in the fossil record of Africa. The diagnostic light microscopy (LM)- and scanning electron microscopy (SEM)-based features of the pollen provide sufficient support to assign the fossils to a distinct lineage within the Loranthaceae. Based on the taxonomic affiliation to extant taxa the palaeophytogeographic signals and palaeoecological aspects of these fossil grains are discussed and potential host taxa are suggested from the currently known palaeo-palynoflora.

## Material and methods

The sedimentary rock containing the fossil Loranthaceae pollen is from core sample #114755 collected at Saldanha Bay, South Africa. The sediments are believed to be of earliest Miocene age. A Chattian to early Miocene age for the Saldanha Bay deposits is suggested on the base of the dinoflagellate indicator taxa *Distatodinium craterum* Eaton, *Chiropteridium lobospinosum* Gocht, *Homotryblium plectilum* Drugg et Loeblich Jr. as well as *Impagidinium paradoxum* (Wall 1967) Stover et Evitt 1978 (see details in Roberts et al. [2017] including supplements). For a full geological, stratigraphic, palaeontological and palaeoenvironmental background of this locality/core see Roberts et al. (2017). The sedimentary rock sample was processed and fossil pollen grains extracted according to the protocol outlined in Grímsson et al. (2008). The fossil Loranthaceae pollen grains were investigated both by LM and SEM using the single grain method as described in Zetter (1989). The description of fossil Loranthaceae pollen includes diagnostic features observed both in LM and SEM. Pollen terminology follows Punt et al. (2007; LM) and Hesse et al. (2009; SEM). Loranthaceae fossil material (SEM stubs) from Saldanha Bay, South Africa, are stored in the collection of the Department of Palaeontology, University of Vienna, Austria under the accession numbers IPUW 7513/211 and IPUW 7513/216.

## Systematic palaeontology

The fossil pollen described here falls within the variation of Pollen Type B defined by Grímsson et al. (2018). Pollen of this type is oblate (to various degrees), triangular to trilobate in polar view and shows a ±psilate sculpturing in LM. Usually, further sculpture details are not observed in LM, but some pollen grains show a clear exine thickening or thinning at the pole and along the colpi or in the mesocolpium. The pollen is syn(3)colpate, see figure 1 in Grímsson et al. (2018) for a schematic drawing. Since the fossil pollen grains described here show combining features known from four extant Loranthaceae genera (see later), we classify this fossil taxon as a morphotype (MT) named after the locality where the pollen occurs.

**Figure 1.**
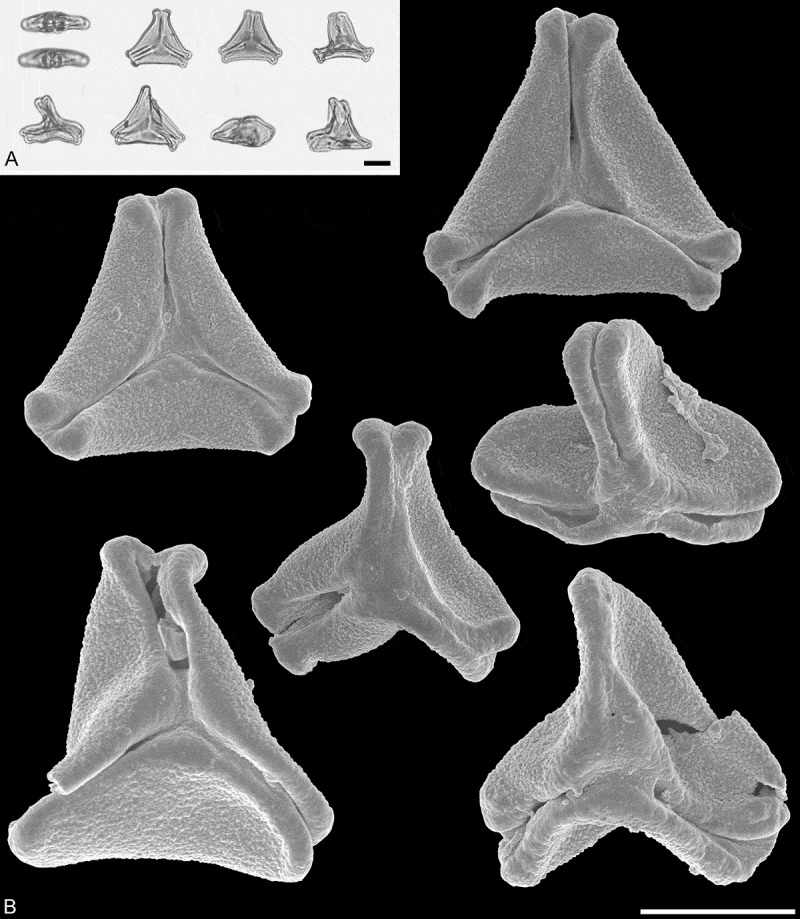
LM (**A**) and SEM (**B**) micrographs of fossil Loranthaceae pollen from the early Miocene of Africa. **A.** Saldanha morphotype (MT) pollen grains in equatorial and polar view. Note triangular intercolpial nexine thickenings in polar area. **B.** Saldanha MT pollen grains in polar view. Equatorial apices are obcordate to T-shaped and the margo is psilate or partly granulate and with triangular protrusions in polar area. Scale bars – 10 µm (A, B).

### Family Loranthaceae Juss.Tribe Lorantheae Rchb.Saldanha MT, aff. Lorantheae*Figures 1*–*3*

*Description*. — Pollen, oblate, concave-triangular to trilobate in polar view, elliptic in equatorial view, equatorial apices obcordate to T-shaped; size small, polar axis 8.8–12.5 µm long in LM, equatorial diameter 20–25 µm in LM, 15–22 µm in SEM; syn(3)colpate; exine 0.8–1.0 µm thick, nexine thinner than sexine (LM), triangular intercolpial nexine thickenings in polar area (LM); tectate; sculpture psilate in LM, nanoverrucate to granulate in area of mesocolpium in SEM, nanoverrucae 0.2–0.5 µm in diameter, verrucae composed of conglomerate granula; margo well developed, margo psilate or partly granulate, margo with triangular protrusions in polar area (SEM); colpus membrane nanoverrucate and granulate (SEM).

*Remarks*. — Compared to extant pollen this fossil MT shows a suite of features found only within the tribe Lorantheae. This combination of outline, size, colpi arrangement, thickening of nexine (LM), and sculpture observed under SEM is typical for taxa placed in the Subtribes Dendropthinae Nickrent & Vidal-Russell (e.g. *Tolypanthus, Dendrophthoe*), Scurullinae Nickrent & Vidal-Russell (e.g. *Taxillus*), and partly Emelianthinae Nickrent & Vidal-Russell (*Phragmanthera*). Both *Tolypanthus maclurei* (Merr.) Danser and *Dendrophthoe pentandra* (L.) Miq. pollen is very similar to the Saldanha MT (see Table II). The *Tolypanthus maclurei* pollen (see figure 38 in Grímsson et al. 2018) is usually larger than the fossils, and the *D. pentandra* pollen (see figure 36 in Grímsson et al. 2018) tends to have a slightly thicker nexine, and wall peculiarities in the polar area are hard to distinguish (LM). Otherwise the pollen of these two taxa is almost identical to the Saldanha MT. Pollen of *Taxillus caloreas* (Diels) Danser (see figure 49 in Grímsson et al. 2018) has a more striking and larger, hexagonal in outline, thickening of nexine in the polar area that differs from that observed in the Saldanha MT. Most other features are comparable to those observed in the fossils. Pollen of *Phragmanthera rufescens* (DC.) Balle (see figure 43 in Grímsson et al. 2018) is more or less identical to the Saldanha MT, except the *P. rufescens* pollen is slightly larger and the exine is thicker (LM).

**Table II.** African fossil morphotype (MT) compared to similar extant pollen and fossil MTs.

	*Tolypanthus maclurei*	*Dendrophthoe pentandra*	*Taxillus caloreas*	*Phragmanthera rufescens* (s.l.)^a^	Saldanha MT (this study)	Changchang MT	Altmittweida MT
**Age/epoch**	Recent	Recent	Recent	Recent	Early Miocene	Middle Eocene (Lutetian-Bartonian)	Late Oligocene-early Miocene (Chattian-Aquitanian)
**Distribution/locality**	East Asia	South, East and Southeast Asia	East Asia	Tropical Africa	Saldanha Bay, South Afruca, core sample #114 755	Changchang Basin, close to Jiazi Town, Qiongshan County, Hainan, China	Altmittweida, Saxony, Germany
**P/E ratio**	oblate	oblate	oblate	oblate	oblate	oblate	oblate
**Outline p.v.**	trilobate to straight-triangular	concave-triangular to trilobate	concave-triangular	concave-triangular	concave-triangular to trilobate	concave-triangular to broadly trilobate	convex-triangular
**Outline eq. v.**	elliptic	elliptic	elliptic	elliptic	elliptic		emarginate
**Equatorial apices**	obcordate	obcordate	obcordate	T-shaped	obcordate to T-shaped	broadly rounded	broadly obcordate
**P in LM (µm)**	8.3–15.8	13.3–15	11.7–15	15–18.3	8.8–12.5		4.4–5.5
**E in LM (µm)**	25–30	21.7–25.8	23.3–30	26.7–31.7	20–25	21.1–24.4	14.4–17.8
**Aperture**	syn(3)colpate	syn(3)colpate	syn(3)colpate	syn(3)colpate	syn(3)colpate	syn(3)colpate	syn(3)colpate
**Exine thickness in LM (µm)**	0.8–1.3	1.1–1.3	1.0–1.3	1.1–1.4	0.8–1.0	0.9–1.1	0.9–1.1
**Wall peculiarities**	triangular intercolpial thickening of nexine in polar area	sexine partly reduced in polar area, colpi widening to a small field	hexagonal nexine thickening in polar area	triangular intercolpial thickening of nexine in polar area	triangular intercolpial thickening of nexine in polar area	rhombic structures (opercula) covering equatorial apices	intercolpial nexine thickening at pole, sexine partly reduced in polar area
**Sculpture (SEM)**	nanoverrucate to granulate	nanoverrucate to granulate	nanoverrucate to granulate	nanoverrucate to granulate	nanoverrucate to granulate	granulate	nano- to microverrucate to granulate
**Type and size of sculpture elements (µm)**	verrucae 0.2–0.5 (−0.8)	verrucae 0.2–0.6	verrucae 0.1–0.5	verrucae 0.1–0.6	verrucae 0.2–0.5		verrucae 0.2–1.3
**Margo (SEM)**	well developed, psilate or partly granulate, with triangular protrusions in polar area	well developed, psilate or partly granulate to nanoverrucate, with triangular protrusions in polar area	well developed, psilate with few nanoverrucae or granula in polar area	well developed, psilate or partly granulate	well developed, psilate or partly granulate, with triangular protrusions in polar area	well developed, psilate	psilate to microverrucate, granulate
**Colpus membrane (SEM)**	nanoverrucate and granulate	nanoverrucate and granulate	nanoverrucate and granulate	nanoverrucate and granulate	nanoverrucate and granulate	granulate	nanoverrucate and granulate

Note: Distribution of extant taxa from Qui and Gilbert (2003) and Polhill and Wiens (1998). Pollen morphology of extant taxa summarised from Grímsson et al. (2018). Pollen morphology of fossil mophotypes summarised from Grímsson et al. (2017). ^a^ *Phragmanthera rufescens* has been widely applied as an aggregate for tropical African *Phragmanthera*. According to Polhill and Wiens (1998) *P. rufescens* is only known from Guinée and the Casamance region of southern Senegal, but the sample figured in Grímsson et al. (2018) is from Cameroon and might therefore represent *P. kamerunensis* or another *Phragmanthera* species.

Despite the number of fossil Loranthaceae pollen reported so far only few grains/types have been studied using SEM (see Grímsson et al. 2017). Of those studied using SEM only two MTs, the Changchang MT form the middle Eocene of China, and the Altmittweida MT from the late Oligocene–early Miocene of Germany (Table II; see also Grímsson et al. 2017), indicate a possible lineage relation (Lorantheae) with the African fossils. The broadly rounded apices, the rhombic structures covering equatorial apertures, and the merely granulate sculpture clearly distinguishes the Chinese Changchang MT from the African Saldanha MT (see Table II). Still, the minute sculpture and the basic form of the Changchang MT also link it to the Lorantheae, especially to the Scurrulinae (*Taxillus, Scurulla*) and Amyeminae (*Amyema*), and Grímsson et al. (2017, p. 21) described this pollen MT as ‘a Scurrulinae pollen with an *Amyema*-like margo’. Therefore, the Changchang MT most likely belongs to an extinct or ancestral Lorantheae lineage related to the core Lorantheae. The emarginate outline in equatorial view, the reduced sexine in the polar area, the microverrucate sculpture in SEM, and the pollen size clearly distinguishes the German Altmittweida MT from the African Saldanha MT. Extant pollen very similar to the Altmittweida MT can be found in two extant species of Lorantheae, *Amyema gubberula* Danser and *Helixanthera kirkii* (Oliv.) Danser. It is therefore also likely that the Altmittweida MT belongs to a lineage related to the core Lorantheae.

## Discussion

### The African Lorantheae fossils in a global (time and space) context

The fossil pollen record of Loranthaceae (e.g. *Gothanipollis*) recently summarised by Grímsson et al. (2017) shows that the family had a worldwide distribution already during the Eocene, with representatives found in South America, North America, Europe, East Asia, and Australasia. Based on this palaeo-phytogeographic pattern it is most likely that Loranthaceae were also present in Africa during that time. The lack of fossil Loranthaceae pollen in the African record should be considered an artefact caused primarily by preparation techniques and study methods, or palynologists working on African material not knowing this typical *Gothanipollis* type. Accepting this, the dispersal of Loranthaceae into Africa might have occurred in the Southern Hemisphere before the final phases of the Gondwana breakup (Late Cretaceous) or in the Northern Hemisphere via Asia (early Eocene). Unfortunately, the majority of Eocene fossil Loranthaceae pollen found in the Southern Hemisphere (South America, Australasia) has mostly been studied using LM only (e.g. Romero & Castro 1986; Raine et al. 2011) and is therefore of very limited use for interfamilial segregation. In a molecular phylogenetic context (see figure 2 in Grímsson et al. 2018) the present African Loranthaceae show a closer relation to South, Southeast and East Asian lineages than any other, and are clearly most distantly related to American Loranthaceae. It is interesting, based on pollen morphology, that the earliest Miocene fossils from Saldanha Bay suggest the same close relation to Asian taxa (Table II) and ‘no’ relation to any of the American lineages. Grímsson et al. (2017) established that several major lineages of Loranthaceae were present during Eocene in the Northern Hemisphere, with records including representatives of extinct or ancestral lineages with affinities to both root-parasitic genera (*Nuytsia*/Nuytsieae) and epiphytic lineages (Lorantheae, Psittacantheae, *Notanthera*, Elytrantheae). In this scenario, it seems more likely that the ancestor(s) of the fossils described here and the current African lineages dispersed into Africa from Asia (northern route) during the Eocene. For now, dispersal into Africa in the Southern Hemisphere during the final phases of the Gondwana breakup cannot be ruled out. If some Loranthaceae were dispersed via a southern route then those lineages became extinct in Africa during the Cainozoic.

**Figure 2.**
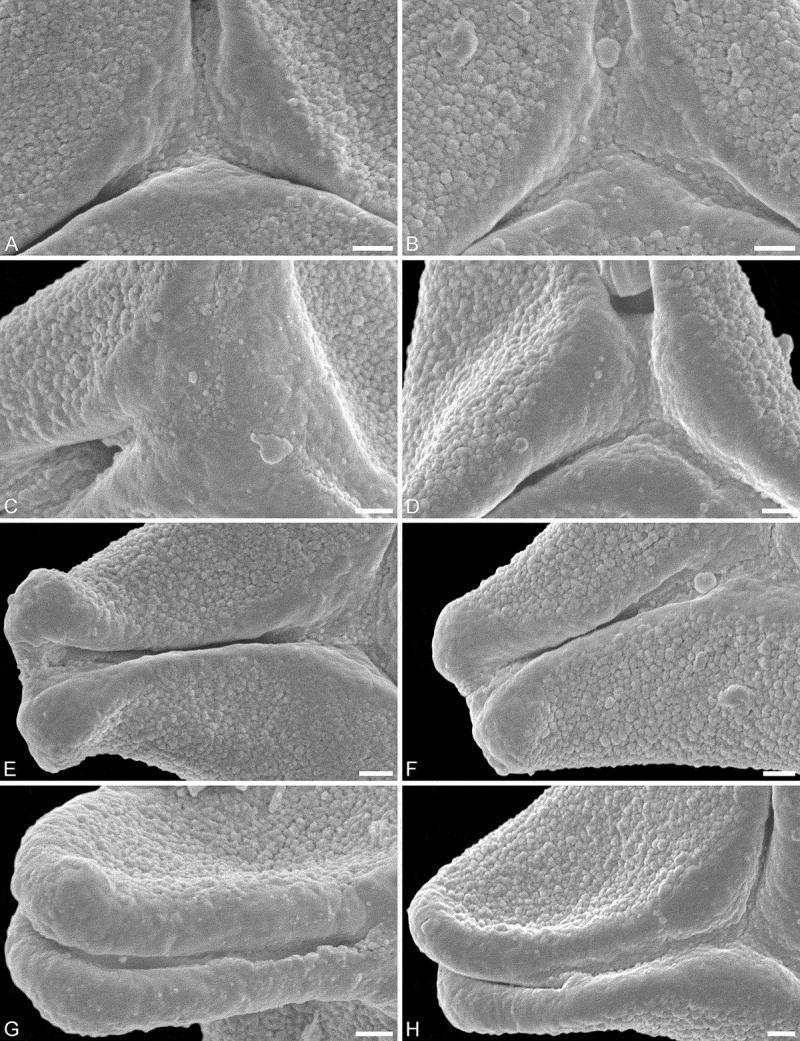
SEM micrographs of fossil Loranthaceae pollen from the early Miocene of Africa. **A–D.** Close-ups of central polar area showing margo with triangular protrusions in polar area. **E–H.** Close-ups of apex showing obcordate to T-shaped apices, and psilate or partly granulate margo. Scale bars – 1 µm (A–H).

**Figure 3.**
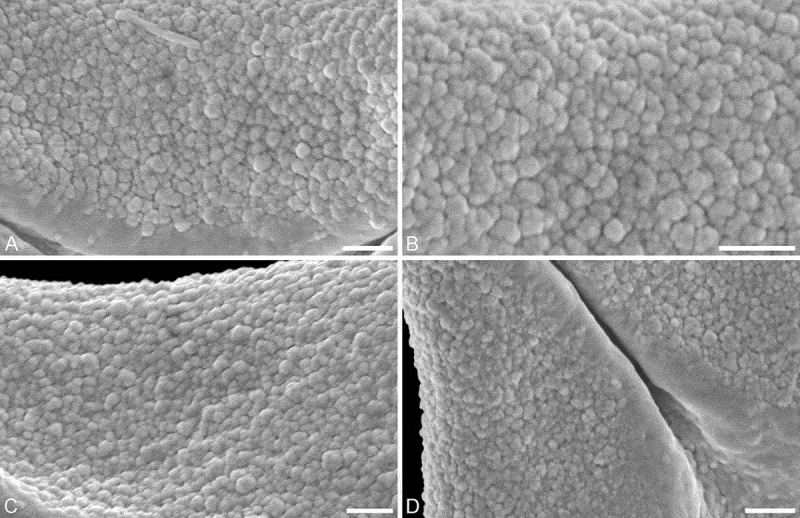
SEM micrographs of fossil Loranthaceae pollen from the early Miocene of Africa. **A–D.** Close-ups of mesocolpium showing nanoverrucate to granulate sculpture (SEM). Scale bars – 1 µm (A–D).

### Time of origin and divergence of African Loranthaceae lineages

Fossil constrained molecular dating by Grímsson et al. (2017) suggests that *Tupeia* (A-type pollen) and Loranthaceae with B-type pollen diverged in the early Eocene (~50 Ma). A primary radiation is believed to have followed shortly thereafter involving the formation of both ‘New World’ (root parasites, Elytrantheae, Psittacantheae) and ‘Old World’ (Lorantheae) clades. Crown group radiation in the Lorantheae is then believed to have started at the latest in the late Eocene (≥ 38 Ma), with a second major radiation taking place ~10 million years later (latest in the Oligocene) involving among others the ‘Old World’ core Lorantheae (Grímsson et al. 2017). Unfortunately, there are no current African records from the Eocene or Oligocene so far, but one could expect to find Loranthaceae pollen showing morphology characteristic for the core crown Lorantheae (e.g. Amyeminae, Dendropthinae) in such samples. Still, the Saldanha MT suggests that until the earliest Miocene pollen producing Loranthaceae in Africa (at least southern Africa) still had the basal Lorantheae pollen form. More diverged lineages/genera must therefore have evolved no earlier than during the latest part of the early Miocene. The age and pollen morphology of the Saldanha MT fit perfectly with the suggested core crown group radiation of Lorantheae and the alleged formation of extant lineages/modern genera lasting until the middle Miocene (≥ 9 Ma; see figure 9 in Grímsson et al. 2017). Fossil pollen showing derived features within the Lorantheae, e.g. Emelianthinae and Tapinanthinae, are most likely to be found in sediments younger than earliest Miocene.

### Ecology and potential hosts of the Saldanha MT

Loranthaceae are currently found in all parts of Africa except the Sahara desert where there is little vegetation. They occur in various habitats, ranging from sea-level to mountain tops, in grasslands as well as rainforests and semi-deserts. Their only requirements seem to be the presence of suitable host plants and dispersal mechanisms (e.g. birds) to carry them between hosts (e.g. Visser 1981; Polhill & Wiens 1998). It is hard to pinpoint the preferred host of a fossil taxon and if it had a narrow (specialist) or wide (generalist) host range. Based on the available host ranges of recent African Loranthaceae (Table II; Tables S1–S21 in Supplemental data) it seems that most of the genera are generalists and parasitising many species/genera/families. The fossil palyno-assemblage containing the Saldanha MT is extremely taxon rich (Roberts et al. 2017) and composing pollen from at least 150 different angiosperms verified using SEM (Grímsson et al. unpublished data). Many of the fossil pollen types belong in families that are known to be parasitised by recent African Loranthaceae. These include Anacardiaceae, Asteraceae, Casuarinaceae, Euphorbiaceae, Fabaceae, Myrtaceae, Oleaceae, Proteaceae, Santalaceae, Sapindaceae, Sapotaceae, and the gymnosperm family Podocarpaceae. Based on the recorded host families listed in Table II, it is likely that every recent Loranthaceae genus would find a suitable host plant in the palaeo-vegetation at Saldanha Bay during the earliest Miocene. The palaeo-vegetation units in the Saldanha Bay region are believed to have been very diverse (Roberts et al. 2017), composed, e.g. of various lowland wetland and marshland forests (mangrove, riparian/swamp) and different mixed evergreen broad-leaved and/or coniferous forests stretching into the surrounding highlands (see figure 13 in Roberts et al. 2017). All potential vegetation units (habitats) recorded by Roberts et al. (2017) would be suitable for Loranthaceae based on the current vast habitat range of the family in Africa (e.g. Polhill & Wiens 1998).

## Conclusion and outlook

Despite the numerous palaeopalynological investigations on African Cretaceous to Miocene microflora there are no comprehensive high resolution SEM-based studies so far. Even though the potential for studying pollen using combined LM and SEM from African sediments was already established by Coetzee in the 1980s (Coetzee 1981, 1983; Coetzee & Muller 1984; Coetzee & Praglowski 1984, 1988), she only presented a handful of fossil taxa using SEM and no other African palynologist has used this combined method since. Based on the current Loranthaceae fossil record it is very unlikely that the family should be absent from Eocene and Oligocene sediments in Africa. It is more likely that the preparation methods (including sieving) and study techniques (counting up to 300 grains in LM) biased the outcome, or/and when present, the palynologist did not know this distinct pollen type and disregarded it or simply misidentified it. Our combined LM and SEM based study shows that the early Miocene South African Loranthaceae fossils resemble the core Lorantheae (Dendrophae, Scurullae) and the more derived lineages (Tapinanthae, Emelianthae) were not present in this area at the time of accumulation. Molecular and fossil signals suggest that Loranthaceae dispersed into Africa via Asia (northern route) during the Eocene. Also, the recent host range of African Loranthaceae and the palaeo-palynological spectrum suggest that the fossil would have no problems finding a host plant during the early Miocene in the Saldanha Bay region. To fully enlighten the African palaeophytogeographic history of this family, including a more precise ‘time of origin’ in both time and space for the derived lineages, Eocene to Pliocene sediments in other parts of Africa as well as middle Miocene to Pleistocene sediments in South Africa must be screened for Loranthaceae pollen and studied using SEM. Upper Cretaceous to Paleocene sediments (e.g. McLachlan & Pieterse 1978; Partridge 1978; Scholtz 1985; Sandersen et al. 2011) should also be screened for Loranthaceae-type pollen to establish if South American/Australasian basal lineages dispersed into Africa via a southern route, but went extinct during the early Cainozoic.

## Supplemental data

Supplementary files: Tables S1-S21.zip

## References

[CIT0001] Barlow BA. 1990 Biogeographical relationships of Australia and Malesia: Loranthaceae as a model In: Baas P, Kalkman K, Geesink R, eds. The Plant Diversity of Malesia, 273–292. Dordrecht: Kluwer Academic Publishers.

[CIT0002] Barlow BA 1983 Biogeography of Loranthaceae and Viscaceae In: Calder M, Bernhardt P, eds. The Biology of Mistletoes, 19–46. Sydney: Academic Press.

[CIT0003] Caires CS 2012 Estudos taxonômicos aprofundados de *Oryctanthus* (Griseb.). Eichler, *Oryctina* Tiegh, e *Pusillanthus* Kuijt (Loranthaceae). PhD Thesis, Universidade de Brasília, Brazil.

[CIT0004] Caires CS, Gomes-Bezerra KM, Proença CEB 2012 Novos sinônimos e uma nova combinação em *Pusillanthus* (Loranthaceae). Acta Botanica Brasilica 26: 668–674. doi:10.1590/S0102-33062012000300016.

[CIT0005] Caires CS, Gomes-Bezerra KM, Proença CEB 2014 A new combination in *Peristethium* (Loranthaceae) expands the genus’ range into the Amazon-Cerrado ecotone. Acta Amazonica 44: 169–174. doi:10.1590/S0044-59672014000200002.

[CIT0006] Caires CS, Gomes-Bezerra KM, Proença CEB 2017 *Passovia myrsinites* a restablished name including *Oryctina atrolineata* (Loranthaceae). Phytotaxa 313: 285–288. doi:10.11646/phytotaxa.313.3.7.

[CIT0007] Coetzee JA 1981 A palynological record of very primitive angiosperms in Tertiary deposits of the south-western Cape Province, South Africa. South African Journal of Science 77: 341–343.

[CIT0008] Coetzee JA 1983 Intimation on the Tertiary vegetation of southern Africa. Bothalia 14: 345–354. doi:10.4102/abc.v14i3/4.1179.

[CIT0009] Coetzee JA, Muller J 1984 The phytogeographic significance of some extinct Gondwana pollen types from the Tertiary of the southwestern Cape (South Africa). Annals of the Missouri Botanical Garden 71: 1088–1099. doi:10.2307/2399246.

[CIT0010] Coetzee JA, Praglowski J 1984 Pollen evidence for the occurrence of *Casuarina* and *Myrica* in the Tertiary of South Africa. Grana 23: 23–41. doi:10.1080/00173138409428875.

[CIT0011] Coetzee JA, Praglowski J 1988 Winteraceae pollen from the Miocene of the southwestern Cape (South Africa). Grana 27: 27–37. doi:10.1080/00173138809427730.

[CIT0012] Dean WRJ, Midgley JJ, Stock WD 1994 The distribution of mistletoes in South Africa: Pattern of species richness and host choice. Journal of Biogeography 21: 503–510. doi:10.2307/2845654.

[CIT0013] Didier DS, Ndongo D, Jules PR, Desiré TV, Henri F, Georges S, Akoa A 2008 Parasitism of host trees by the Loranthaceae in the region of Douala (Cameroon). African Journal of Environmental Science and Technology 2: 371–378.

[CIT0014] Dlama TT, Oluwagbemileke AS, Enehezeyi AR 2016 Mistletoe presence on five tree species of Samaru area, Nigeria. African Journal of Plant Science 10: 16–22. doi:10.5897/AJPS2015.1335.

[CIT0015] Dzerefos CM, Witkowski ETF, Shackleton CM 2003 Host-preference and density of woodrose-forming mistletoes (Loranthaceae) on savanna vegetation, South Africa. Plant Ecology 167: 163–177. doi:10.1023/A:1023991514968.

[CIT0016] Feuer SM, Kuijt J 1978 Fine structure of mistletoe pollen I. Eremolepidaceae, *Lepidoceras*, and *Tupeia* . Canadian Journal of Botany 56: 2853–2864. doi:10.1139/b78-341.

[CIT0017] Feuer SM, Kuijt J 1979 Pollen evolution in the genus *Psittacanthus* Mart. Fine structure of mistletoe pollen II. Botaniska Notiser 132: 295–309.

[CIT0018] Feuer SM, Kuijt J 1980 Fine structure of mistletoe pollen III. Large-flowered neotropical Loranthaceae and their Australian relatives. Annals of the Missouri Botanical Garden 72: 187–212. doi:10.2307/2399176.

[CIT0019] Feuer SM, Kuijt J 1985 Fine structure of mistletoe pollen VI. Small-flowered neotropical Loranthaceae. Annals of the Missouri Botanical Garden 72: 187–212. doi:10.2307/2399176.

[CIT0020] Grímsson F, Denk T, Zetter R 2008 Pollen, fruits, and leaves of *Tetracentron* (Trochodendraceae) from the Cainozoic of Iceland and Western North America and their palaeobiogeographic implications. Grana 47: 1–14. doi:10.1080/00173130701873081.

[CIT0021] Grímsson F, Grimm GW, Zetter R 2018 Evolution of pollen morphology in Loranthaceae. Grana 57: 16–116. doi:10.1080/00173134.2016.1261939. PMC5771552 29386990

[CIT0022] Grímsson F, Kapli P, Hofmann C-C, Zetter R, Grimm GW 2017 Eocene Loranthaceae pollen pushes back divergence ages for major splits in the family. PeerJ 5: e3373. doi:10.7717/peerj.3373. 28607837 PMC5466002

[CIT0023] Han R-L, Zhang D-X, Hao G. 2004 Pollen morphology of the Loranthaceae from China. Acta Phytotaxonomica Sinica 42: 436–456.

[CIT0024] Hesse M, Halbritter H, Zetter R, Weber M, Buchner R, Frosch-Radivo A, Ulrich S 2009 Pollen terminology – an illustrated handbook. Vienna: Springer.

[CIT0025] Kuijt J 1988 Revision of *Tristerix* (Loranthaceae). Systematic Botany Monographs 19: 1–61. doi:10.2307/25027693.

[CIT0026] Liu L-F, Qiu H-X 1993 Pollen morphology of Loranthaceae in China. Guihaia 13: 235–245. (in Chinese with English abstract).

[CIT0027] McLachlan IR, Pieterse E 1978 Preliminary palynological results: Site 361, Leg 40, Deep Sea Drilling Project. Initial Reports of the Deep Sea Drilling Project 40: 857–881.

[CIT0028] Nickrent DL 1997–onwards The Parasitic Plant Connection. http://parasiticplants.siu.edu; accessed September 2017.

[CIT0029] Nickrent DL, Dl N, Malécot V, Vidal-Russell R, Der JP 2010 A revised classification of Santalales. Taxon 9: 538–558.

[CIT0030] Ogunmefun OT, Fasola TR, Saba AB, Oripuda OA 2013 The ethnobotanical, phytochemical and mineral analyses of *Phragmanthera incana* (Klotzsch), a species of mistletoe growing on three plant hosts in south-western Nigeria. International Journal of Biomedical Science 9: 37–44. PMC3644413 23675287

[CIT0031] Okubamichael DY, Griffiths ME, Ward D 2013 Reciprocal transplant experiment suggests host specificity of the mistletoe *Agelanthus natalitius* in South Africa. Journal of Tropical Ecology 30: 153–163. doi:10.1017/S0266467413000801.

[CIT0032] Okubamichael DY, Griffiths ME, Ward D 2016 Host specificity in parasitic plants – Perspectives from mistletoes. AoB PLANTS 8: plw69. doi:10.1093/aobpla/plw069. PMC5206351 27658817

[CIT0033] Partridge DA 1978 Palynology of the late tertiary sequence at site 361, Leg 40, Deep Sea Drilling Project. Initial Reports of the Deep Sea Drilling Project 40: 953–961.

[CIT0034] Polhill R, Wiens D 1998 Mistletoes of Africa. Kew: The Royal Botanic Gardens.

[CIT0035] Polhill RM, Wiens D 1999 Loranthaceae. Flora of Tropical East Africa 179: 1–121.

[CIT0036] Punt W, Hoen P, Blackmore S, Nilsson S, Le Thomas A 2007 Glossary of pollen and spore terminology. Review of Palaeobotany and Palynology 143: 1–81. doi:10.1016/j.revpalbo.2006.06.008.

[CIT0037] Qui H, Gilbert MG 2003 Loranthaceae In: Wu ZY, Raven PH, Hong DY, eds. Flora of China, Volume 5, Ulmaceae through Basellaceae, 220–239. Beijing: Science Press.

[CIT0038] Raine JI, Mildenhall DC, Kennedy EM 2011 New Zealand fossil spores and pollen: An illustrated catalogue, 4th edition GNS Science miscellaneous series no. 4 http://data.gns.cri.nz/sporepollen/index.htm; accessed September 2017.

[CIT0039] Roberts DL, Neumann FH, Cawthra HC, Carr AS, Scott L, Durugbo EU, Humphries MS, Cowling RM, Bamford MK, Musekiwa C, MacHutchon M 2017 Palaeoenvironments during a terminal Oligocene or early Miocene transgression in a fluvial system at the southwestern tip of Africa. Global and Planetary Change 150: 1–23. doi:10.1016/j.gloplacha.2017.01.007.

[CIT0040] Roldán FJ, Kuijt J 2005 A new, red-flowered species of *Tripodanthus* (Loranthaceae) from Columbia. Novon 15: 207–209.

[CIT0041] Romero EJ, Castro MT 1986 Material fúngico y granos de polen de angiospermas de la Formación Río Turbio (Eoceno), provincia de Santa Cruz, República Argentina. Ameghiniana 23: 101–118.

[CIT0042] Roxburgh L, Nicolson SW 2005 Patterns of host use in two African mistletoes: The importance of mistletoe-host compatibility and avian disperser behaviour. Functional Ecology 19: 865–873. doi:10.1111/fec.2005.19.issue-5.

[CIT0043] Sandersen A, Scott L, McLachlan I, Hancox J 2011 Cretaceous biozonation based on terrestrial palynomorphs from two wells in the offshore Orange Basin of South Africa. Palaeontologia Africana 46: 21–41.

[CIT0044] Scholtz A 1985 The palynology of the upper lacustrine sediments of the Arnot Pipe, Banke, Namaqualand. Annals of the South African Museum 95: 1–109.

[CIT0045] Veste M 2007 Parasitic flowering plants on *Euphorbia* in South Africa and Namibia. Euphorbia World 3: 5–9.

[CIT0046] Visser J 1981 South African parasitic flowering plants. Cape Town: Juta.

[CIT0047] Wiens D, Tölken HR 1979 Loranthaceae In: Leistner OA, ed. Flora of South Africa, Vol. 10, Part 1. Pretoria: Botanical Research Institute.

[CIT0048] Zetter R 1989 Methodik und Bedeutung einer routinemäßig kombinierten lichtmikroskopischen und rasterelektonenmikroskopischen Untersuchung fossiler Mikrofloren. Courier Forschungsinstitut Senckenberg 109: 41–50.

